# MERS-CoV Found in *Hyalomma dromedarii* Ticks Attached to Dromedary Camels at a Livestock Market, United Arab Emirates, 2019

**DOI:** 10.3390/v15061288

**Published:** 2023-05-30

**Authors:** Pia Weidinger, Jolanta Kolodziejek, Tom Loney, Dafalla O. Kannan, Babiker Mohammed Osman, Tamer Khafaga, Brigitte Howarth, Moayyed Sher Shah, Hessa Mazrooei, Nadine Wolf, Noushad Karuvantevida, Ahmad Abou Tayoun, Alawi Alsheikh-Ali, Jeremy V. Camp, Norbert Nowotny

**Affiliations:** 1 Viral Zoonoses, Emerging and Vector-Borne Infections Group, Institute of Virology, University of Veterinary Medicine Vienna, 1210 Vienna, Austria; pia.weidinger@vetmeduni.ac.at (P.W.); jolanta.kolodziejek@chello.at (J.K.); na_wolf@gmx.at (N.W.); 2 College of Medicine, Mohammed Bin Rashid University of Medicine and Health Sciences, Dubai P.O. Box 505055, United Arab Emirates; tom.loney@mbru.ac.ae (T.L.); hessa.mazrooei@students.mbru.ac.ae (H.M.); noushad.karuvantevida@mbru.ac.ae (N.K.); alawi.alsheikhali@mbru.ac.ae (A.A.-A.); 3 Al Ain City Municipality, Al Ain P.O. Box 1003, United Arab Emirates; dafalla.ahmed@aam.gov.ae (D.O.K.); babiker.osman@aam.gov.ae (B.M.O.); 4 Dubai Desert Conservation Reserve, Emirates Group, Dubai P.O. Box 686, United Arab Emirates; t.khafaga@ksrnr.gov.sa (T.K.); m.shah@ksrnr.gov.sa (M.S.S.); 5 Natural History Museum Abu Dhabi (NHMAD), Department of Culture and Tourism, Abu Dhabi P.O. Box 94000, United Arab Emirates; bhowarth@dctabudhabi.ae; 6 Al Jalila Genomics Center of Excellence, Al Jalila Children’s Specialty Hospital, Dubai P.O. Box 7662, United Arab Emirates; ahmad.tayoun@ajch.ae; 7 Center for Genomic Discovery, Mohammed Bin Rashid University of Medicine and Health Sciences, Dubai P.O. Box 505055, United Arab Emirates; 8 Center for Virology, Medical University of Vienna, 1090 Vienna, Austria; jeremy.camp@meduniwien.ac.at

**Keywords:** MERS-CoV, coronavirus, UAE, dromedary, camel, tick, *Hyalomma dromedarii*

## Abstract

The main mode of transmission of Middle East respiratory syndrome-related coronavirus (MERS-CoV) between dromedaries is likely via the respiratory route. However, there must be other modes to explain how the infection is brought to MERS-CoV-negative closed herds, such as transmission by ticks. Here, we present a study performed at three different locations in the United Arab Emirates (UAE) involving 215 dromedary camels (*Camelus dromedarius*) and the ticks attached to them. We tested the camels and ticks via RT-(q)PCR for the presence of MERS-CoV nucleic acids, as well as flaviviruses that may be present in the region (e.g., Alkhumra hemorrhagic fever virus). Camel sera were additionally analyzed for evidence of previous exposure to MERS-CoV. In total, 8 out of 242 tick pools were positive for MERS-CoV RNA (3.3%; C_t_ 34.6–38.3), 7 of which contained *Hyalomma dromedarii* ticks, and one contained a *Hyalomma* sp. tick (species not identified). All of the virus-positive ticks’ host camels were also positive for MERS-CoV RNA in their nasal swab samples. Short sequences established in the *N* gene region from two positive tick pools were identical to viral sequences from their hosts’ nasal swabs. In total, 59.3% of dromedaries at the livestock market had MERS-CoV RNA in their nasal swabs (C_t_ 17.7–39.5). While dromedaries at all locations were negative for MERS-CoV RNA in their serum samples, antibodies were detected in 95.2% and 98.7% of them (tested by ELISA and indirect immunofluorescence test, respectively). Given the probably transient and/or low level of MERS-CoV viremia in dromedaries and the rather high C_t_ values observed in the ticks, it seems unlikely that *Hyalomma dromedarii* is a competent vector for MERS-CoV; however, its role in mechanical or fomite transmission between camels should be investigated.

## 1. Introduction

Ticks (order Ixodida) are involved in the transmission of a large variety of pathogens due to their blood-feeding behavior in vertebrate hosts. Immature stages of ticks associated with human pathogens often feed on small mammals and birds, and on larger mammals as adults, thereby connecting diverse branches of vertebrates, collecting and spreading pathogens among many different hosts [1]. Ticks, feeding on all classes of terrestrial vertebrates, are second in importance only to mosquitoes as vectors of human pathogens, and are the primary carriers of pathogens with veterinary relevance [2]. Ticks of the *Hyalomma* genus (family Ixodidae) have especially gained attention due to their capability to transmit zoonotic viruses to humans, such as Crimean-Congo hemorrhagic fever orthonairovirus (CCHFV), and occasionally also West Nile virus and Rift Valley fever virus, as well as their ability to transmit several pathogens of veterinary importance, causing theileriosis, babesiosis, and anaplasmosis in various animal species, with high economic impacts [3]. Ticks surpass all other hematophagous arthropods in the diversity of transmitted pathogens, including viruses [4].

At present, at least 160 different tick-borne viruses (TBVs) are known, and newly emerging tick-borne diseases are steadily reported, with some of them constituting a significant threat to human and/or animal health [4]. In recent decades, various TBVs have also re-emerged and/or spread to new areas due to anthropogenic activities, such as tick-borne encephalitis virus and African swine fever virus (ASFV) [4,5,6,7,8]. TBVs are a heterogenous group of arboviruses (arthropod-borne viruses) belonging to several different families. Apart from one exception (ASFV), all TBVs are RNA viruses, and about 25% of them cause diseases, from very serious to less serious and/or infrequently reported [4].

Viral zoonotic diseases that have previously been reported from the Arabian Peninsula include Crimean-Congo hemorrhagic fever (CCHF), Alkhumra hemorrhagic fever (AHF), and Middle East respiratory syndrome (MERS) [9]. CCHFV (family *Nairoviridae*) is a TBV found across Europe, Africa, Asia, and the Middle East [9,10,11,12]. Infection of mammalian livestock, such as camels, sheep, goats, and cattle, by *Hyalomma* ticks has been reported. Transmission to humans may occur through contact with contaminated blood, body fluids, or animal tissues at slaughter, through tick bites or by nosocomial transmission [10,11,13]. While the infection remains asymptomatic in most animals [9], humans can develop severe disease, with a case fatality ratio (CFR) of up to 40% [10,11,13].

Alkhumra hemorrhagic fever virus (AHFV), a flavivirus (family *Flaviviridae*) causing hemorrhagic fever, was first isolated in Saudi Arabia in 1995 [14]. Human cases of AHF have mainly been linked to camels and sheep, although the virus has never been detected in livestock animals, but instead in *Hyalomma dromedarii* ticks in Saudi Arabia, Yemen, and Kuwait [9,15]. The role of mammalian species regarding the propagation and maintenance of AHFV remains unclear. Humans may become infected by consumption of unpasteurized camel milk, direct contact to infected livestock animals and their raw meat, or potentially by mosquito and tick bites [14,16].

Several other of the more than 70 flaviviruses are important human pathogens, such as West Nile virus, yellow fever virus, dengue viruses, Japanese encephalitis virus, tick-borne encephalitis virus, Zika virus, and St. Louis encephalitis virus [17]. These flaviviruses are also transmitted by arthropods, such as mosquitoes and ticks, and can be important veterinary pathogens infecting many different animal species. There has been a remarkable global spread of flaviviruses during the last 70 years [18]. West Nile virus, for instance, which is predominantly transmitted by mosquitoes, but sporadically also by ticks [19,20], was introduced to the American continent in 1999 (lineage 1) [21], to Europe in 2004 (lineage 2) [22], and is also present on the Arabian Peninsula [23]. Other flaviviruses documented to be found in the UAE include Barkedji and Bagaza viruses [24].

In 2012, Middle East respiratory syndrome-related coronavirus (MERS-CoV), a novel zoonotic coronavirus, emerged in humans and was first reported in a man in Saudi Arabia [25]. Since then, MERS-CoV has been reported in humans in 27 countries, with 84% of cases occurring in Saudi Arabia, and a CFR of 36%. Worldwide, 2600 laboratory-confirmed cases and 935 MERS-related deaths have been reported to WHO (until October 2022) [26]. Similar to other coronaviruses, it is assumed that MERS-CoV originated from bats; however, the predominating reservoir and source of infection in humans are believed to be dromedary camels (*Camelus dromedarius*) [27,28,29,30,31,32,33,34,35,36]. In endemic regions, herds of dromedaries have high proportions of animals actively shedding the virus, depending on seasonal and environmental factors, and seropositivity typically approaches 100% in older camels [33,37]. Despite the fact that these animals usually remain asymptomatic or develop only mild symptoms due to MERS-CoV infection, they can shed substantial amounts of virus from the respiratory tract [29,31,38,39].

Hitherto, MERS-CoV nucleic acids have neither been detected in sera or whole blood samples of dromedaries, sheep, goats, or cattle, nor in ticks [15,38,40,41,42,43]. However, the exact transmission cycle and the animals involved remain uncertain [28,44]. In closed camel herds, i.e., herds without contact to animals from outside the herd, MERS-CoV infections may persist for a certain time period and then cease for a while, until suddenly cases start to rise again without any apparent introduction event. This may be due to the time of calving during winter months, with a subsequently rising incidence once a critical number of vulnerable young animals is reached [45]. However, it remains to be elucidated by which transmission mode(s) the infection is brought to MERS-CoV-negative closed herds. Recently, we explored the possible role of wild rodents in this matter, but did not find any indications for their involvement [46]. The potential role of ticks in the transmission of MERS-CoV remains to be further investigated, as the mechanisms allowing TBVs to switch from ticks to vertebrate hosts as well as viral persistence in various environments are not yet fully understood [4].

In 2019, we performed a study in the United Arab Emirates (UAE) on diverse samples from dromedary camels and other livestock animals, including ticks attached to them. From this field study, we previously reported the transmission activity of CCHFV in dromedary camels and camel ticks (*Hyalomma dromedarii*) at a livestock market, and the transmission activity (including possible spillover) of MERS-CoV at the same market [11,13,43]. In the present study, using the same cohort, we tested the camels and their associated ticks for the presence of MERS-CoV and flaviviruses.

## 2. Materials and Methods

### 2.1. Sampling

This study was part of an ongoing public health surveillance program in the UAE. Nasal swab and blood samples were collected during two field seasons (March/April and October 2019) from 215 dromedaries at three locations: the largest national livestock market situated in the emirate of Abu Dhabi (April 2019, n = 50; October 2019, n = 90); a desert wildlife reserve with camels used for tourism (April 2019, n = 60); and a family-owned farm in Dubai with camels raised mainly for breeding, racing, and trading (March 2019, n = 15). The spatial distribution of the pens at the market and the pens with MERS-CoV nucleic acid-positive dromedaries were shown previously by satellite image and schematic diagram [43].

Before blood collection and swab sampling, each animal was searched thoroughly (~2 min) for attached ticks. In total, we collected 314 adult ticks and 33 nymphs in March and April 2019, and 214 adult ticks and 4 nymphs in October 2019 (0–5 ticks/camel). Cross-contamination during sampling was prevented by changing gloves after each animal, and ticks were not collected from the nasal areas of camels to avoid contamination with nasal discharge.

Nasal swabs were transferred to tubes containing a virus inactivation solution (DNA/RNA Shield; ZymoResearch, Irvine, CA, USA). Serum was obtained from whole blood samples by centrifugation. All samples, including ticks, were then stored at −80 °C at the laboratory of the College of Medicine, Mohammed Bin Rashid University of Medicine and Health Sciences, Dubai, UAE, before shipment on dry ice to the University of Veterinary Medicine, Vienna, Austria, for virological and serological investigations.

### 2.2. Serological Investigations

Sera from dromedaries were tested for MERS-CoV-specific antibodies by ELISA and indirect immunofluorescence test (IIFT). For this purpose, samples were thawed at 32 °C, vortexed and centrifuged for 2 min at 400× *g*, before performing the assays according to the manufacturer’s instructions (Anti-MERS-CoV ELISA Camel (IgG) and Anti-MERS Coronavirus IIFT Camel (IgG); both from Euroimmun, Lübeck, Germany). Photometric measurement of the color intensity generated by ELISA was performed using a Tecan GENios Microplate Reader (Tecan Group, Männedorf, Switzerland), and IIFT slides were evaluated using an Olympus IX70 Fluorescence Microscope (Olympus, Tokyo, Japan).

### 2.3. Processing of Tick Samples

Frozen adult ticks were morphologically identified on an ice-cold plate under a stereomicroscope using various keys. The vast majority were *Hyalomma dromedarii* ticks (98.7%, 521/528), three were *H. scupense*, and four were not further specified ticks of the genus *Hyalomma*. Frozen ticks were processed by making a parasagittal section with a sterile scalpel, and tick halves were pooled per tick species and individual host (≤5 per pool; 242 pools in total), homogenized in a bead mill (TissueLyser II; QIAGEN, Hilden, Germany) in phosphate-buffered saline, and centrifuged before adding 2× (concentrated) DNA/RNA Shield. Thereafter, samples were again vortexed, centrifuged, and then frozen at −80 °C for at least one hour before extraction.

### 2.4. Nucleic Acid Extraction and RT-(q)PCR Assays

Homogenized tick samples were thawed, vortexed, and centrifuged for 3 min at 3500× *g*. Sera were mixed with 2× (concentrated) DNA/RNA Shield and briefly centrifuged, as were the nasal swab samples (already in DNA/RNA Shield). Subsequently, 140 or 200 µL of each supernatant were subjected to automatic nucleic acid extraction employing QIAamp Viral RNA Mini QIAcube Kit on a QIAcube device (for 12 samples) or QIAamp 96 Virus QIAcube HT Kit on a QIAcube HT robot (plate format; all from QIAGEN) according to the manufacturer’s instructions.

All extracts were screened by reverse transcription (real-time) polymerase chain reaction (RT-(q)PCR) for the presence of MERS-CoV and AHFV nucleic acids. In addition, we used a ‘universal’ flavivirus RT-PCR that has been demonstrated to detect many members of the *Flaviviridae* family.

For initial MERS-CoV screening, all extracts were tested by RT-qPCR targeting the open reading frame (ORF) 1a [47]; samples resulting in a positive signal were also tested by RT-qPCR targeting ORF 1b to confirm putative positives [48]. In addition, samples were screened by AHFV RT-qPCR [49]. All RT-qPCRs were performed using primers and probes at concentrations of 0.5 µM each, employing Quantabio qScript XLT 1-Step RT-qPCR ToughMix (Quantabio, Beverly, MA, USA) under the following conditions: 50 °C for 15 min, 95 °C for 2 min, and 45 cycles of 95 °C for 15 s and 60 °C for 30 s. All RT-qPCRs were performed on Applied Biosystems 7500 Real-Time PCR system (Thermo Fisher Scientific, Waltham, MA, USA) or qTOWER³ G (Analytik Jena, Jena, Germany). Cycle threshold (C_t_) values ≤ 39.5 were considered putative positive, according to the internal validation of these assays.

In addition, conventional semi-nested RT-PCR in the nucleocapsid (N) gene of MERS-CoV was performed on RT-qPCR-positive samples [47], and all samples were screened for flaviviruses by RT-PCR using ‘universal’ flavivirus S/AS2 primers [50]. RT-PCRs were performed using QIAGEN OneStep RT-PCR Kit (QIAGEN) under the following conditions: 50 °C for 30 min; 95 °C for 15 min; 50 cycles of 94 °C for 30 s, 60 °C for 30 s, and 72 °C for 30 s; and a final elongation step at 72 °C for 7 min. The second round of the MERS-CoV semi-nested PCR was performed using QIAGEN Fast Cycling PCR Kit (QIAGEN), under the following conditions: 95 °C for 5 min; 50 cycles of 96 °C for 5 s, 60 °C for 5 s, 68 °C for 20 s; and final elongation at 68 °C for 1 min. All RT-(q)PCRs included negative and positive controls.

### 2.5. Sequencing and Phylogenetic Analysis

Conventional RT-PCR products were subjected to automatic gel electrophoresis on QIAxcel Advanced System (QIAGEN). Nucleotide sequences were obtained by Sanger sequencing using Mix2Seq Kits (Eurofins Genomics, Ebersberg, Germany), identified by BLAST search (https://blast.ncbi.nlm.nih.gov/Blast.cgi, accessed on 29 March 2023), and aligned using BioEdit Sequence Alignment Editor version 7.2.5. The phylogenetic tree was created in MEGA X [51], using the Jukes–Cantor and p-distance methods with 1000 bootstrap replicates.

## 3. Results

### 3.1. Tick Samples

In total, 8 of the 242 tick pools were positive for MERS-CoV RNA (3.3%), 5/142 collected in March/April and 3/100 from October (C_t_ values 34.6–38.3). Seven of those pools contained *H. dromedarii* ticks, and one pool contained *Hyalomma* sp. ticks. All of their host camels were also positive for MERS-CoV RNA in their nasal swab samples (Table 1). Neither AHFV nor other flaviviruses could be detected in any of the tick pools.

**Figure 1:**
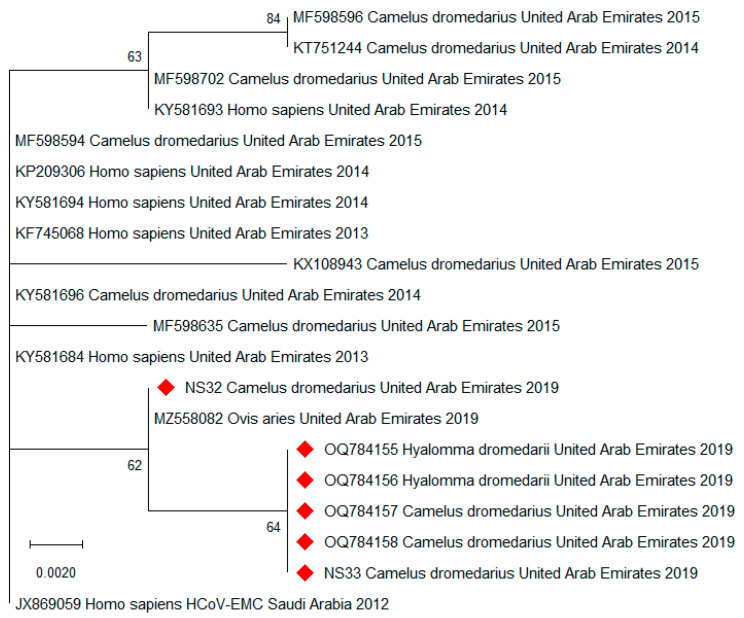
Maximum likelihood phylogenetic tree of 20 partial MERS-CoV sequences. The 192 bp fragments of the sequences generated in this study (OQ784155-OQ784158, NS32, and NS33; NS = nasal swab; red diamonds) were analyzed together with sequences from humans, camels (*Camelus dromedarius*), and a sheep (*Ovis aries*) from the UAE from 2013 to 2019, as well as with the sequence of the first human MERS case detected in Saudi Arabia in 2012 (HCoV-EMC). For each sequence, the corresponding GenBank accession number (where available), host species, country of origin, and collection year are indicated. Horizontal lines represent the genetic distances according to the scale. The tree was inferred using the Jukes–Cantor substitution model over 1000 bootstrap replicates (percentages are displayed at the nodes).

By conventional semi-nested RT-PCR in the N gene region of MERS-CoV, we were able to establish short sequences (221–224 bp) from two of the positive tick pools but were unsuccessful in amplifying this region from the rest of the positive pools. Multiple sequence alignment revealed 100% nucleotide identity between these sequences and the virus sequences obtained from the host nasal swabs. By BLAST search, the closest nucleotide identities (99.6%) were found to MERS-CoV strains from humans and camels in Saudi Arabia from 2015 to 2019. All four sequences were deposited to GenBank (accession numbers OQ784155-OQ784158) and aligned with other sequences from humans, camels, and a sheep (*Ovis aries*) from the UAE from 2013 to 2019, as well as with the sequence of the first human MERS case detected in Saudi Arabia in 2012 (Supplemental Figure S1). Reference sequences from the UAE were selected from all available years and host species, and identical sequences were omitted. From this alignment, a phylogenetic tree was constructed based on 192 bp fragments of MERS-CoV (Figure 1).

The sequences derived from tick pools and their dromedary hosts (OQ784155-OQ784158) were identical. Compared to the sequence of the first human MERS case, all sequences from 2019 differed by two single-base substitutions at most, while the remaining bases were highly conserved in this short MERS-CoV genome region. Phylogenetic analysis showed clustering of the sequences from 2019 relative to the sequences established in the years before.

### 3.2. Dromedary Samples

From the 215 camel nasal swabs, 42 in April and 41 in October were positive for MERS-CoV nucleic acid (38.6%, C_t_ 17.7–39.5); all of which were collected at the market (59.3%, n = 140). All serum samples from the 215 camels were negative for MERS-CoV RNA, but the vast majority of dromedaries at all three locations had antibodies to MERS-CoV in their sera (95.2% and 98.7%, tested by ELISA and IIFT, respectively). Neither AHFV nor other flaviviruses could be detected in any of the samples. Of note, all positive dromedary and tick samples were collected at the livestock market, whereas all samples from the farm and the wildlife reserve tested negative for all viruses including CCHFV [11,13].

## 4. Discussion

In this study, we investigated a cohort of camels and associated ticks in the UAE, where we previously documented transmission of MERS-CoV and CCHFV [11,13,43]. We detected MERS-CoV RNA in eight pools of ticks collected from camels at a livestock market that were confirmed to also be MERS-CoV RNA-positive in their nasal swab samples, and we found that the virus sequences associated with the ticks were identical.

A hematophagous arthropod able to transmit pathogens via blood feeding is termed a biological vector. Vector competence in this regard is an arthropod’s innate ability to incorporate, maintain, and transmit a microbe [1]. Competent vectors of TBVs are capable of being infected even if viremia in the infected host is transient or undetectable. As ticks typically feed on one host per life stage, TBVs must be able to survive molting (transstadial survival). Some TBVs may also be transmitted vertically from one generation to the next, or sexually from males to females [1].

TBVs, in contrast to tick-specific viruses, are able to replicate in invertebrate as well as vertebrate cells, as they depend on the transmission between ticks and vertebrate hosts for survival. Here, the ticks enable ‘biological transmission’ in contrast to simple ‘mechanical transmission’, in which the virus does not replicate within tick tissue [52]. Therefore, the successful isolation of a virus from a tick, especially when it has recently fed on a host, does not imply that this species is a competent vector, as the virus may only be contained in the bloodmeal [52]. Crude associations regarding pathogens found in engorged ticks have contributed to numerous misconceptions [53]. Thus, our data need to be interpreted very cautiously, as the detection of MERS-CoV in ticks does not imply that they are able to transmit the virus while feeding on the next host. The fact that all camel sera were negative for MERS-CoV nucleic acid, although 59.3% of dromedaries at the market shed the virus via the upper respiratory tract, contradicts even marginal viremia, and this may rule out biological transmission.

However, *Hyalomma dromedarii* ticks are very mobile, actively ’chasing’ dromedaries and crawling on the camels’ bodies in order to find the most suitable place for undisturbed blood feeding or mating partners [54]. Therefore, it is possible that ticks are also moving across the nasal area of an infected dromedary, thereby becoming contaminated with viral particles in the camel’s nasal discharge. Alternatively, other selected feeding locations may allow the ticks to become contaminated with the virus (e.g., near the anus in contact with excreta, as ticks prefer humid skin regions [55]), and the virus may be transmitted via dromedaries’ grooming behaviors (self or social). It is known that ticks play a role as mechanical vectors of some viruses (e.g., lumpy skin disease virus [54]), and the role of contaminated ticks as fomites remains to be seen. Obviously, there are many gaps in fully explaining how ticks may facilitate the spread of MERS-CoV. However, based on our observations, transmission via ticks cannot be entirely excluded, especially when considering the high viral loads shed by camels during acute infection [29,31,38,39]. Further studies on the role of ticks and the environmental stability of MERS-CoV are required to determine, for instance, how long mechanical vectors may remain infectious.

We found a remarkable difference regarding MERS-CoV-positive dromedaries between the three different sampling sites, with 59.3% of acutely infected animals at the market and not a single current infection at the family farm or the wildlife reserve. This high proportion of infections at the market is in line with other studies from livestock markets (27–59%) [56,57,58], compared to much lower ratios of dromedaries that tested positive at farms (3.7–11.1%) [29,30]. On average, about 1500 animals, including camels, sheep, goats, and cattle, are traded daily at this market, primarily from all over the UAE, but also from neighboring countries, such as Oman and Saudi Arabia. Animals usually stay 1 to 30 days until they are sold to farms or slaughtered on site. Animals with elevated stress levels due to transportation over long distances, crowded conditions, and other factors have an increased risk of developing diseases. Together with the length of stay at the market, this seems to provide an ideal situation for infections to spread among animals, and eventually also humans. Human infections were beyond the scope of this study; however, there have been epidemiological links between camel markets and human disease [34,35]. Precautions are in place to minimize infections at the market, such as grouping camels from the same owner in the same pens, frequently replacing sand in the pens, and regularly turning the soil in order to eliminate ticks. In addition, severely tick-infested animals are not allowed to enter the market. At the time of sampling, acaricides were applied to all other livestock animals (except during the last three days before slaughter), but only to a limited extent to camels. To the best of our knowledge, dromedaries meanwhile receive a complete acaricide treatment, which is expected to reduce TBV infections, provided that effective agents are used and applied strategically, especially from March to June when *H. dromedarii* ticks are most abundant [3,55]. To prevent the spread of MERS-CoV from dromedaries to humans, further measures should be implemented, such as restricting dromedary movement without prior testing for active infections, promoting the use of personal protective equipment by camel handlers, raising awareness about the potential risk accompanying the consumption of unpasteurized camel milk and urine, and developing effective vaccines [33].

In contrast to MERS-CoV, it is well-established that CCHFV can be considered a TBV, as it is found in many tick species and can even persist in them for their entire lifespan, although only a few species act as vectors and reservoirs [1]. Of note, one of the MERS-CoV-positive tick pools from April also tested positive for CCHFV RNA (C_t_ 23.9). As previously reported, in total, five tick pools were CCHFV-positive (2.1%; C_t_ 22.1–32.3), two in April and three in October [11,13]. However, all sera of camels with associated positive ticks were negative for both MERS-CoV and CCHFV nucleic acids. At the market, one serum from April and two sera from October tested positive for CCHFV RNA (1.4%; C_t_ 36.7–37.6) [11,13]. In contrast, all sera collected at the wildlife reserve and the farm tested negative, although antibodies to CCHFV were found in 77.4% of all camels (tested by ELISA), which indicates that this virus is widespread in the region as well [11]. Despite the high incidence of active infections with MERS-CoV in dromedaries, MERS-CoV RNA was not detected in any of their sera. However, antibodies to MERS-CoV were found in the vast majority of dromedaries at all three locations (95.2% and 98.7%, tested by ELISA and IIFT, respectively). Thus, even though both viruses are widely distributed, no active infections were found at the wildlife reserve or farm, further emphasizing the epidemiological role of large livestock markets.

The main limitation of our study is that we only report molecular detection of MERS-CoV RNA, but did not test whether the virus was infectious due to biosafety restrictions. Additionally, the concentration of virus in the tick pools was quite low (C_t_ 34.6–38.3), and we were only able to sequence the partial N gene from two of the tick pools. Thus, the informative value of our phylogenetic analysis is limited by the number and length of the sequences (192 bp). However, we previously established five complete *N* gene sequences from MERS-CoV-positive dromedaries from the same sample cohort (MZ558077-MZ558081) [43], and many complete MERS-CoV genome sequences derived from camels from the same market in 2015 were previously published by others [58]. As MERS-CoV is—contrary to SARS-CoV-2—a highly conserved virus species, strains isolated months apart from the same location are often identical [33], which is in accordance with the observation that our newly established sequences are almost identical to all available reference sequences from the UAE. However, we acknowledge that the comparative value of our phylogenetic analysis is further limited due to this conserved nature of MERS-CoV. Our findings may also differ from others, in that we did not surface decontaminate the ticks prior to homogenization [41]. However, contamination during collection of the ticks was prevented by changing gloves after each animal was sampled, and not collecting ticks from the nasal area. Moreover, the sampling procedure per animal was carried out as follows: first the animal was searched for ticks, then the blood sample was taken, and finally (because most unpleasant for the camel) the nasal swabs were taken. Therefore, the accidental transfer of virus from nasal swabs to ticks can also be excluded. However, further investigations involving viral cultures are required to elucidate how ticks may facilitate the spread of MERS-CoV.

## 5. Conclusions

We found MERS-CoV RNA in 8 out of 242 tick pools (3.3%), all collected from dromedary camels at a livestock market in the UAE. All of their host camels were also positive for MERS-CoV RNA in their nasal swab samples, but negative in their serum samples. Partial MERS-CoV sequences derived from nasal swabs of two of these dromedaries were identical to the MERS-CoV sequences of the *Hyalomma dromedarii* ticks attached to them. Collectively, the most probable explanation for these observations is that the ticks became contaminated with the virus while in contact with their dromedary host. Although it seems unlikely that ticks are competent vectors for this virus, their potential role in MERS-CoV transmission should be explored in further studies, preferably including attempts to isolate the virus from tick specimens.

## Figures and Tables

**Table 1:** RT-qPCR results of tick pools positive for MERS-CoV nucleic acid.

Collection Date	Tick Species	Tick No. and Stages (M/F/nymph) *	C_t_ Tick Pools	C_t_ Camel Nasal Swabs
28 April 2019	*H. dromedarii*	2/0/0	35.3–37.9	32.8–37.4
28 April 2019	*H. dromedarii*	1/1/0	36.5–37.8 ^†^	30.3–34.2 ^†^
28 April 2019	*H. dromedarii*	1/1/0	34.8–36.7 ^†^	31.1–34.8 ^†^
28 April 2019	*H. dromedarii*	2/0/2	34.6–36.8	29.0–33.9
28 April 2019	*H. dromedarii*	2/0/0	36.4–37.7	34.6–36.1
12 October 2019	*H. dromedarii*	2/0/0	35.8–36.1	32.8–36.8
12 October 2019	*Hyalomma* sp.	1/0/0	36.5–37.1	33.2–38.5
12 October 2019	*H. dromedarii*	2/2/0	38.3	32.6–37.6

* M (adult male), F (adult female); ^†^ sequence available in GenBank.

## Data Availability

The sequences that were established in this study were submitted to GenBank database (acc. no. OQ784155-OQ784158). The corresponding author is happy to share any other raw data upon reasonable request.

## Supplementary Materials

The following supporting information can be downloaded at: https://www.mdpi.com/article/10.3390/v15061288/s1, Figure S1: Multiple sequence alignment of partial MERS-CoV sequences from *Hyalomma dromedarii* tick pools compared to reference sequences.
