# Supplementary material for: MERS-CoV Found in Hyalomma dromedarii Ticks Attached to Dromedary Camels at a Livestock Market, United Arab Emirates, 2019
Source: Viruses. 2023 May 30;15(6):1288. doi: 10.3390/v15061288 (PMC10303736; doi:10.3390/v15061288)
Supplement: Supplementary file 1 [file viruses-15-01288-s001.zip › viruses-2398637-supplementary.pdf]

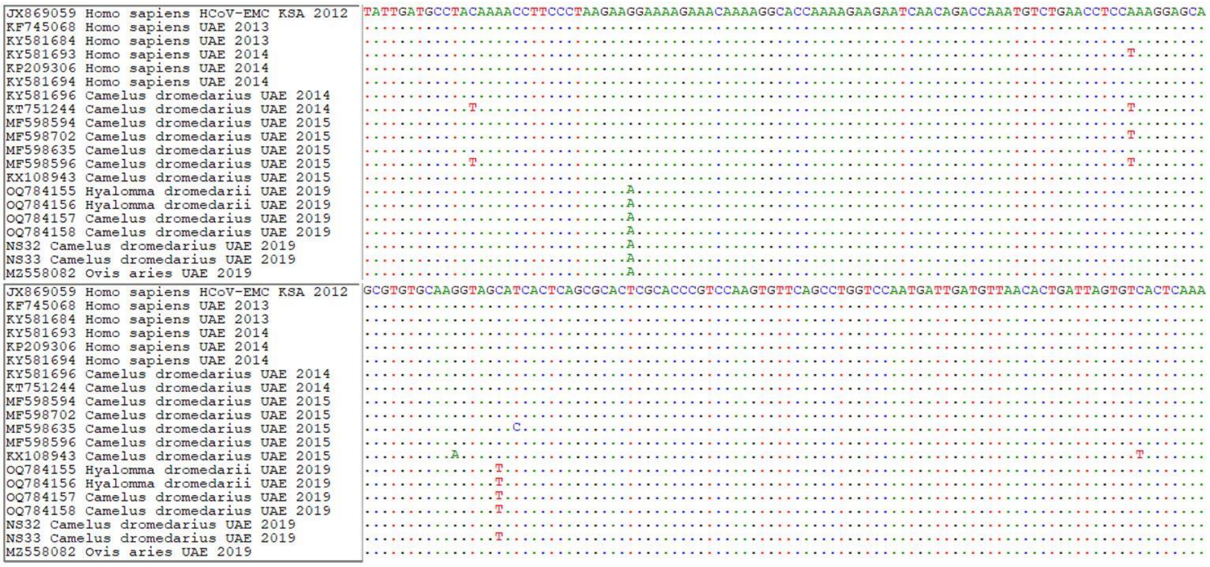

Supplemental Figure S1. Multiple sequence alignment of partial MERS-CoV sequences from *Hyalomma dromedarii* tick pools compared to reference sequences. For each sequence, the corresponding GenBank accession number (where available), host species, country of origin and collection year are indicated. Sequences generated in this study (OQ784155-OQ784158, NS32 and NS33; NS = nasal swab) were aligned with sequences from humans, camels (*Camelus dromedarius*) and a sheep (*Ovis aries*) from the UAE from 2013-2019. The sequence of the first human MERS case detected in Saudi Arabia in 2012 (HCoV-EMC) is shown at the top of each panel, and substitutions relative to this reference strain are indicated by letters, using a dot to represent a conserved site. The figure was produced in BioEdit Sequence Alignment Editor version 7.2.5.
